# Cost-effectiveness of 2-[^18^F]FDG-PET/CT versus CE-CT for response monitoring in patients with metastatic breast cancer: a register-based comparative study

**DOI:** 10.1038/s41598-023-43446-7

**Published:** 2023-09-28

**Authors:** Mohammad Naghavi-Behzad, Oke Gerke, Annette Raskov Kodahl, Marianne Vogsen, Jon Thor Asmussen, Wolfgang Weber, Malene Grubbe Hildebrandt, Kristian Kidholm

**Affiliations:** 1 Department of Clinical Research, University of Southern Denmark, Odense, Denmark; 2 Department of Nuclear Medicine, Odense University Hospital, Kløvervænget 15, 5000 Odense, Denmark; 3 Centre for Personalized Response Monitoring in Oncology, Odense University Hospital, Odense, Denmark; 4 Department of Oncology, Odense University Hospital, Odense, Denmark; 5 Open Patient Data Explorative Network (OPEN), Odense University Hospital, Odense, Denmark; 6 Department of Radiology, Odense University Hospital, Odense, Denmark; 7 Department of Nuclear Medicine, Technical University of Munich, Munich, Germany; 8 Department of Radiology, Memorial Sloan Kettering Cancer Center, New York, USA; 9 Centre for Innovative Medical Technology, Odense University Hospital, Odense, Denmark

**Keywords:** Breast cancer, Health care economics, Medical imaging

## Abstract

We evaluated the cost-effectiveness of 2-[^18^F]FDG-PET/CT compared to CE-CT for response monitoring in metastatic breast cancer (MBC) patients. The study included 300 biopsy-verified MBC patients treated at Odense University Hospital (Denmark). CE-CT was used in 144 patients, 83 patients underwent 2-[^18^F]FDG-PET/CT, and 73 patients received a combination of both. Hospital resource-based costs (2007–2019) were adjusted to the 2019 level. The incremental cost-effectiveness ratio (ICER) was calculated by comparing average costs per patient and gained survival with CE-CT. During a median follow-up of 33.0 months, patients in the 2-[^18^F]FDG-PET/CT group had more short admissions (median 6 vs. 2) and fewer overnight admissions (5 vs. 12) compared to the CE-CT group. The mean total cost per patient was €91,547 for CE-CT, €83,965 for 2-[^18^F]FDG-PET/CT, and €165,784 for the combined group. The ICER for 2-[^18^F]FDG-PET/CT compared to CE-CT was €-527/month, indicating gaining an extra month of survival at a lower cost (€527). 2-[^18^F]FDG-PET/CT was more cost-effective in patients with favorable prognostic factors (oligometastatic or estrogen receptor-positive disease), while CE-CT was more cost-effective in poor prognosis patients (liver/lung metastases or performance status ≥ 2 at baseline). In conclusion, our study suggests that 2-[^18^F]FDG-PET/CT is a cost-effective modality for response monitoring in metastatic breast cancer.

## Introduction

International guidelines do not provide clear recommendations on modality of choice for monitoring response to treatment in patients with metastatic breast cancer (MBC)^1,2^, but contrast-enhanced computed tomography (CE-CT) is often used in clinical practice based on its general availability^2^. CE-CT has a sensitivity ranging between 57 and 77% for diagnosing distant metastases^3,4^. 2-deoxy-2-[^18^F]fluoro-D-glucose PET/CT positron emission tomography with integrated computed tomography (2-[^18^F]FDG-PET/CT) has, however, shown sensitivity of almost 100% for the diagnosis of distant metastases in multiple studies^3,4^. Previous studies have also shown higher sensitivity of 2-[^18^F]FDG-PET/CT than CE-CT for evaluating disease response and progression, while CE-CT more frequently reports stable disease^5,6^. In recent studies on patients with MBC from our group, 2-[^18^F]FDG-PET/CT could detect the first progression five-six months earlier than CE-CT on average^6,7^. 2-[^18^F]FDG-PET/CT has also been shown to be a superior predictor of progression-free and disease-specific survival than CE-CT in MBC patients^8^. These findings suggest that 2-[^18^F]FDG-PET/CT may improve the clinical management when used for response monitoring in MBC patients^1,7^.

The cost of using 2-[^18^F]FDG-PET/CT for response monitoring of metastatic disease has been a concern for healthcare providers^9,10^. However, no economic evaluation has compared the two diagnostic modalities (CE-CT vs. 2-[^18^F]FDG-PET/CT) to determine how efficiently they use healthcare resources to monitor MBC patients^11,12^. This calls for a careful evaluation of the potential cost-effectiveness of using 2-[^18^F]FDG-PET/CT for MBC patients^5,10,13^.

In our recent register-based comparison, we showed an average survival benefit of 14 months for MBC patients undergoing 2-[^18^F]FDG-PET/CT for response monitoring compared with patients monitored with CE-CT^7^. In the current study, we aimed to investigate the cost-effectiveness of applying 2-[^18^F]FDG-PET/CT versus CE-CT in response monitoring of the same population of MBC patients. Specifically, we investigated the cost-effectiveness of using 2-[^18^F]FDG-PET/CT versus CE-CT over time by adding cost data to the results from our prevoiusly reported survival analysis of MBC patients^7^. The cost-effectiveness analysis was performed for subgroups of patients with favorable prognostic factors such as estrogen receptor-positive disease, oligometastatic disease, and high performance status upon diagnosis as well as unfavorable prognostic factors such as liver or lung metastases.

## Material and methods

This single-centre, register-based study was conducted at the Department of Nuclear Medicine, Odense University Hospital (Denmark) between November 2018 and June 2022. Patients were identified from a previously reported study^7^, and this is an additional cost-effictiveness analysis.

### Patient selection and study groups

**Figure 1:**
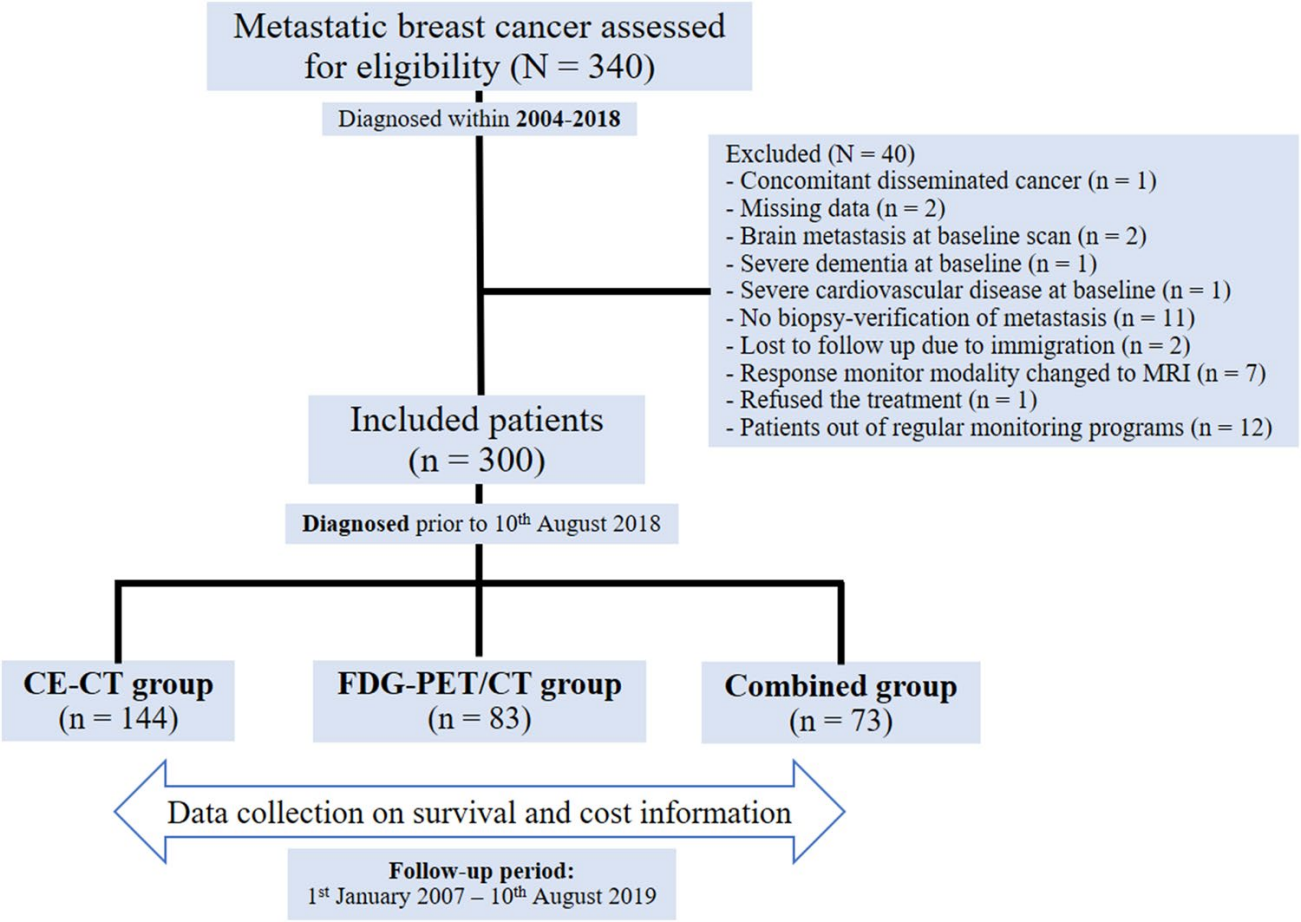
Flowchart of patient selection and categorization into the study groups. (CE-CT, contrast-enhanced computed tomography; FDG-PET/CT, ^18^Fluorodeoxyglucose positron emission tomography with integrated computed tomography; MRI, magnetic resonance imaging).

Women diagnosed with MBC between 2004 and 2018 were eligible for this study. All patients were treated at the Department of Oncology, and the imaging for response monitoring was conducted at the Department of Nuclear Medicine and/or Radiology at Odense University Hospital (Denmark). Inclusion criteria were biopsy-verified distant relapse or de novo MBC patients (biopsy verification of primary tumor or distant metastases along with disseminated disease at baseline scan); available baseline scan and and at least one follow-up scan; use of either 2-[^18^F]FDG-PET/CT, CE-CT, or a combination of the two as the main response monitoring modality; regular clinical follow-up; and available information on health-related costs. Exclusion criteria were other known disseminated malignancy; brain metastasis at baseline scan; change of response monitoring modality to magnetic resonance imaging (MRI); acute cardiovascular disease or severe dementia at the time of inclusion; missing clinical or cost data; lost to follow-up due to emigration; and declining of treatment. The CE-CT and 2-[^18^F]FDG-PET/CT scans were performed with imaging intervals of 9–12 weeks based on a standard response monitoring protocol^14^. The study groups were similar to those of the previous study^7^, and the patients were categorized into three groups (Fig. 1) based on the imaging modality used for response monitoring: CE-CT group (*n* = 144), 2-[^18^F]FDG-PET/CT group (*n* = 83), and the combined group (*n* = 73). The clinical information was extracted from the patients’ medical files, as reported in more detail in the previous study^7^.

### The Danish healthcare system

Danish healthcare is financed by a national, tax-based insurance system and operates on the principles of free and equal access to healthcare services for all residents. The out-of-pocket costs of healthcare services for cancer patients is almost negligible in Denmark as the national healthcare system covers the costs related to treatment and monitoring. All healthcare-related costs are routinely registered and are accessible for research projects according to the principles of the Danish Data Protection Agency^15,16^.

### Collection of health-related cost data

This economic evaluation follows the approaches described by the Consolidated Health Economic Evaluation Reporting Standards (CHEERS) guideline^17^. The economic perspective was that of the hospital sector, and the study uses data on the use of hospital resources.

Resources related to the treatment of patients during the follow-up period (January 1, 2007 until August 10, 2019) were registered. The hospital-based resources consisted of (1) admissions, which included short-stay admissions (under 24 h) and overnight stays; (2) outpatient visits; (3) laboratory tests; (4) imaging modalities; (5) received treatments; (6) surgeries, including minimal biopsies; and (7) palliative care provided by public sector.

The patients’ use of hospital resources was estimated based on hospital contacts recorded in the National Patient Register^18^. Price estimates for use of inpatient and outpatient hospital care by each patient were derived from the National Patient Register and were based on the Diagnosis Related Groups (DRG/DAGS) used in the Danish reimbursement system^19^. According to the Danish DRG system, a single DRG rate is used for patients who have several services during single visit/admission^16,20^. Registered costs from different years were adjusted to the cost levels of 2019 (last day of follow-up) according to the Danish healthcare inflation list^21^. All rates were valued in Danish kroner (DKK) and are reported in Euros (€) at the exchange rate of 7.45 DKK/€. Table 1 shows examples of routine DRG rates related to the diagnosis, treatment, and monitoring of MBC patients.

**Table 1:** Examples of costs in the Danish healthcare system based on Diagnosis Related Group (DRG) codes (2019 level)^21^.

Characteristics	DRG code	Cost (€)
Breast cancer diagnosis package	09MA08	4719
Visit at department of oncology	BVAA3	238
Basic hospital bed price (per day)	“Sengedag”	279
Imaging		
2-[^18^F]FDG-PET scan	36PR03	1203
2-[^18^F]FDG-PET with integrated CT scan	36PR02	1549
Contrast-enhanced CT scan	30PR06	269
MRI scan	30PR02	392
Treatment planning for anti-cancer treatments		
Vinorelbine + Pertuzumab/Trastuzumab	27MP22	3237
Paclitaxel ± Carboplatin	27MP21	2308
Eribulin + antibody therapy	27MP19	4605
Antibody therapy	27MP26	2312
Fulvestrant + radiotherapy (1–2 fractions)	27MP23	2834
Radiation therapy (standard protocol)	27MP15	1031
Anti-cancer treatments		
Tamoxifen + visit at department	34PR06	856
Fulvestrant + Pegfilgrastim	34PR05	1258
Palbociclib + Letrozol	BWHA442	1645
Letrozol + visit at department of oncology	BWHC12	856
Radiation therapy		
Complete program of radiation therapy (at least 5 fractions)	27MP01	14,557
Radiation therapy (two fractions)	27MP03	812
Radiation therapy (single fraction)	27MP04	416
Invasive procedures		
Biopsy with fine needle aspiration (FNA)	09PR04	645
Needle biopsy on lymph nodes with ultrasound guide	05PR02	652
Mastectomy (with/without lymph node dissection)	09MP04-6	1869–4923

### Statistical analyses

Continuous data are presented using median (range) and mean ± standard deviation. Graphical displays comprised box plots in which indivually indicated data points were either larger than the 3rd quartile plus the interquartile range or smaller than the 1st quartile minus the interquartile range. Frequencies and respective percentages are given for categorical variables. Kruskal–Wallis and Chi-squared tests served exploratory assessement of inter-group differences in continuous and categorical variables, respectrively. The mean-based incremental cost-effectiveness ratio (ICER) was calculated as the ratio between the mean cost (€) per patient and the gained median survival (months) within the CE-CT versus 2-[^18^F]FDG-PET/CT by considering death or end of study period as the censoring event for both survival and cost analyses. The start point for survival analysis of patients diagnosed before 2007 was set to January 1, 2007 (left-censoring). The median-based ICER was calculated as a supplementary analysis^22^. The overall monthly cost was calculated from the total registered cost (adjusted to the 2019 price) divided by the follow-up time for each patient and was compared between the study groups. The logrank test was used to explore differences in 1-, 2-, 5-, and 10-year survival, censoring appropriately (Supplementary Material 1). The statistical significance level was set at 0.05. All statistical analyses were done using STATA/IC software (version 16.1, StataCorp, College Station, USA).

### Subgroup and sensitivity analyses

We re-analyzed the ICER for subgroups of patients with the most important prognostic parameters selected from the previous study^7^. Oligometastatic disease was defined as patients with fewer than five metastatic lesions in a single organ^23^. Performance status was based on the World Health Organization scale^24^. As an additional subgroup, we analyzed the ICER for patients who had estrogen receptor-positive, HER2 receptor-negative, non-oligometastatic disease. A further sensitivity analysis was conducted on a subgroup of patients from the CE-CT group (83/144) who were matched by age, performance status, year of diagnosis, and number of involved organs to patients from the 2-[^18^F]FDG-PET/CT group. Matching was performed using the STATA procedure psmatch2.

### Ethical approval and informed consent

The study protocol was approved by the Danish Patient Safety Authority (Ethics permission code: 3-3013-2448/1), and permission to register data from the patients’ electronic medical files and to access information on health-related costs was granted by the Ethics Committee of the Region of Southern Denmark. Due to the nature of this retrospective study and the preserved anonymity of patients, informed consent was waived by Ethics Committee of the Region of Southern Denmark (permission code: 17/29,850).

## Results

The median follow-up period was comparable for the CE-CT (29.7 months; range: 3.7–107.0) and 2-[^18^F]FDG-PET/CT (30.1 months; range: 2.4–124.3) groups, but was longer for the combined group (44.3 months; range: 7.4–151.0). A summary of the most important prognostic parameters and the survival times within the study groups are shown in Supplementary Material 1.

### Inpatient and outpatient visits

Table 2 provides an overview of inpatient and outpatient visits and details of received treatments and imaging modalities during the follow-up period. The frequency of outpatient visits was comparable between study groups, but the 2-[^18^F]FDG-PET/CT group had considerably more short-stay admissions (*P* < 0.001) and fewer overnight hospital stays (*P* = 0.002) than the CE-CT and combined groups. The patients in all groups received a similar number of scans per three months during the response monitoring period.

**Table 2:** Overview of inpatient and outpatient visits, response monitoring scans, and treatments during the follow-up period.

Characteristics	Study groups			P-value
	CE-CT (*n* = 144)	2-[^18^F]FDG-PET/CT (*n* = 83)	Combined (*n* = 73)	
Inpatient and outpatient visits, median (range)				
Number of outpatient visits	96.5 (16–346)	96 (19–420)	155 (40–556)	< 0.001
Number of short-stay admissions	2 (0–15)	6 (1–25)	3 (0–20)	< 0.001
Hospital overnight stays	12 (0–103)	5 (0–118)	11 (0–167)	0.002
Total number of scans, median (range)	11 (3–36)	11 (3–36)	18 (5–51)	< 0.001
Number of scans per 3 months*, median (range)	1.2 (0.4–3.6)	1.1 (0.3–5.8)	1.2 (0.2–2.7)	0.4
Number of received treatment lines, median (range)	3 (1–8)	2 (0–8)	3 (1–9)	< 0.001
Duration (months) of treatment courses, median (range)	6.8 (0.50–49.3)	7.7 (0.50–76.7)	9.3 (1.4–105)	0.01
Exposure of patients to treatment categories, frequency (%)				
Chemotherapy	99 (68.8)	45 (54.2)	57 (78.1)	0.006
Endocrine therapy	107 (74.3)	61 (73.5)	53 (72.6)	0.97
Anti-HER2 therapy	13 (9.0)	7 (8.4)	17 (23.3)	0.009
CDK4/6 inhibitors	19 (13.2)	19 (22.9)	12 (16.4)	0.18
Bone-target therapy	105 (72.9)	60 (72.3)	52 (71.2)	0.97

CE-CT, contrast-enhanced computed tomography; 2-[^18^F]FDG-PET/CT, ^18^Fluorodeoxyglucose positron emission tomography with integrated computed tomography; HER2, human epidermal growth factor receptor 2.

*Total number of performed response-monitoring scans (CE-CT, 2-[^18^F]FDG-PET/CT and MRI) adjusted by follow-up period.

### Overall and department-specific costs

An overview of total costs and cost distribution during the follow-up period is shown in Table 3. Total cost, hospital stay cost, and the cost related to the Department of Oncology activities and imaging modalities (costs directly related to clinical management) were lower in the 2-[^18^F]FDG-PET/CT group than in either the CE-CT group or the combined group. The mean total cost in the 2-[^18^F]FDG-PET/CT group was €7581 lower than in the CE-CT group (*P* = 0.59). There were no differences between the CE-CT and 2-[^18^F]FDG-PET/CT groups in the costs related to departments other than oncology (€7353 vs. €6925) or the departments related to surgical specialties (€4181 vs. €4799).

**Table 3:** Overview of costs distribution during follow-up period^*^

Characteristics	Study groups			*P*-value
	CE-CT (*n* = 144)	2-[^18^F]FDG-PET/CT (*n* = 83)	Combined (*n* = 73)	
Total costs during whole follow-up period				
Mean ± standard deviation	91,547 ± 65,220	83,965 ± 57,390	165,784 ± 130,193	–
Median (range)	73,667 (9585–394,275)	65,685 (17,390–341,934)	110,621 (30,269–585,875)	< 0.001
Hospital stay costs				
Mean ± standard deviation	19,015 ± 20,391	15,014 ± 18,088	25,736 ± 25,934	–
Median (range)	13,858 (0–123,600)	9281 (0–92,778)	18,665 (0–138,807)	< 0.001
Distribution of total costs to different departments**, mean (range)				
Oncology department + imaging costs	75,068 (4037–388,732)	67,573 (5,810–323,655)	140,403 (18,690–561,358)	< 0.001
Other departments*** costs	7353 (0–77,708)	6925 (0–78,266)	11,984 (0–157,898)	0.07
Surgical departments costs	4181 (0–57,522)	4799 (0–33,470)	8,064 (0–39,067)	< 0.001
Palliative care costs	4946 (0–54,306)	4667 (0–47,538)	5,333 (0–92,061)	0.55
Costs within different time periods****, median (range)				
Costs within first 3 months	8975 (608–59,538)	11,584 (610–37,441)	10,038 (1,641–58,178)	0.41
Costs during 4–6th months	6764 (352–51,295)	6813 (333–33,584)	6,444 (337–62,718)	0.51
Costs during 7–12th months	11,836 (991–55,800)	8830 (464–48,458)	12,731 (378–81,243)	0.24
Costs within 2nd year (13–24th months)	19,382 (991–82,767)	16,167 (1628–99,528)	27,170 (2,361–91,958)	0.01
Costs during 3rd–5th years	34,645 (416–213,402)	23,395 (3475–220,289)	67,431 (1,462–369,744)	< 0.001
Costs during 6–10th years	25,605 (1,226–148,436)	18,927 (4562–97,529)	81,168 (4,834–204,579)	0.02

CE-CT, contrast-enhanced computed tomography; 2-[^18^F]FDG-PET/CT, ^18^fluorodeoxyglucose positron emission tomography with integrated computed tomography.

*All costs are reported in Euros.

**All departments excluding departments of oncology, nuclear medicine, and radiology and departments related to surgical specialties.

***The analyses was done only on available patients in each specific time and the patients with zero cost were excluded.

### Time-related costs

**Figure 2:**
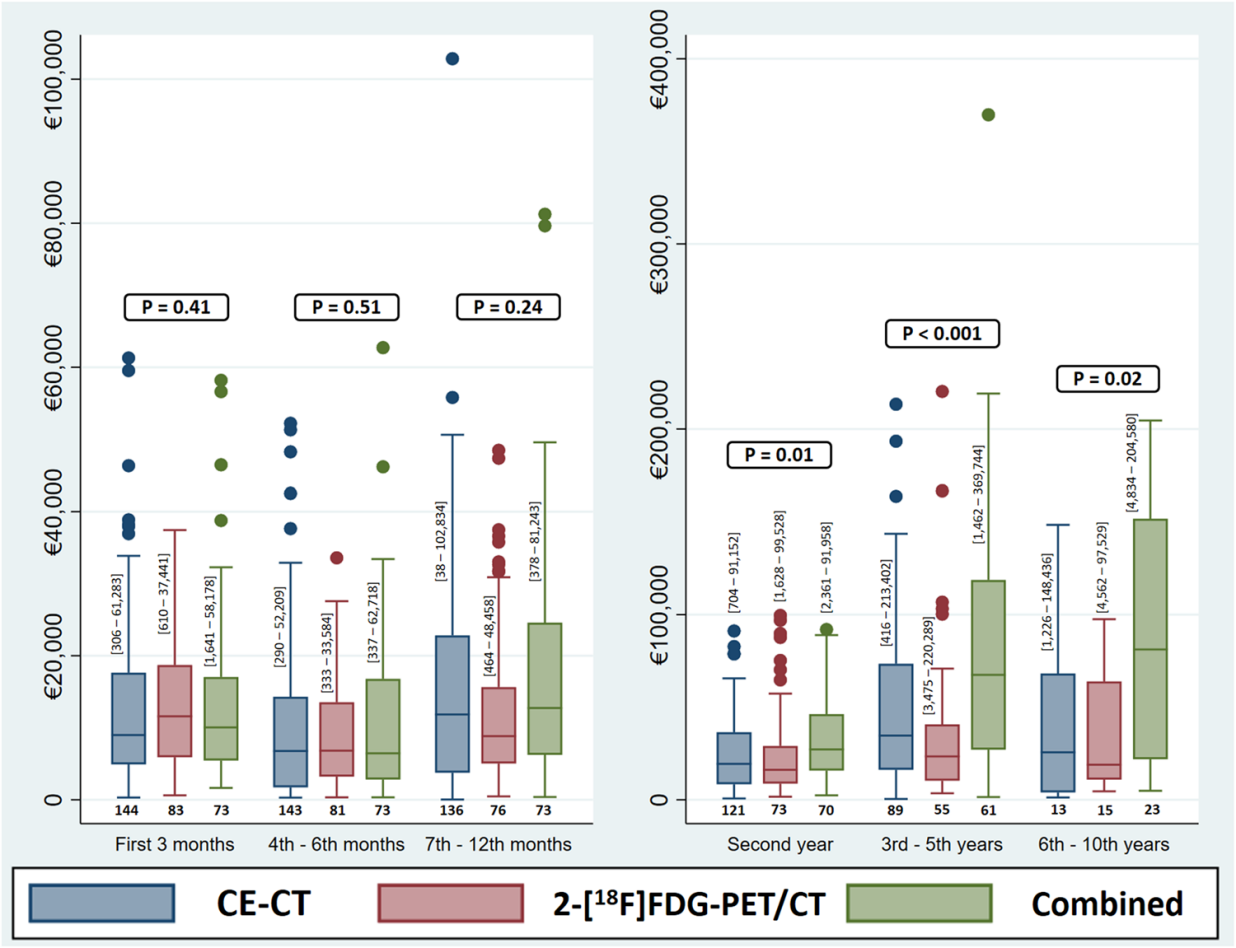
Box plots to compare the costs for each study group during different follow-up periods. Patients with zero cost in each time period were excluded from comparisons. The numbers in brackets close to each box plot represent the range of cost in each specific group (CE-CT, contrast-enhanced computed tomography; FDG-PET/CT, ^18^Fluorodeoxyglucose positron emission tomography with integrated computed tomography).

The cost within the first three months was higher in the 2-[^18^F]FDG-PET/CT group than in the CE-CT and the combined groups (*P* = 0.41), whereas the cost was lower in the 2-[^18^F]FDG-PET/CT group during the 7–12th months (*P* = 0.24), the second year (*P* = 0.01), and for the longer follow-up period (Table 3 and Fig. 2).

**Figure 3:**
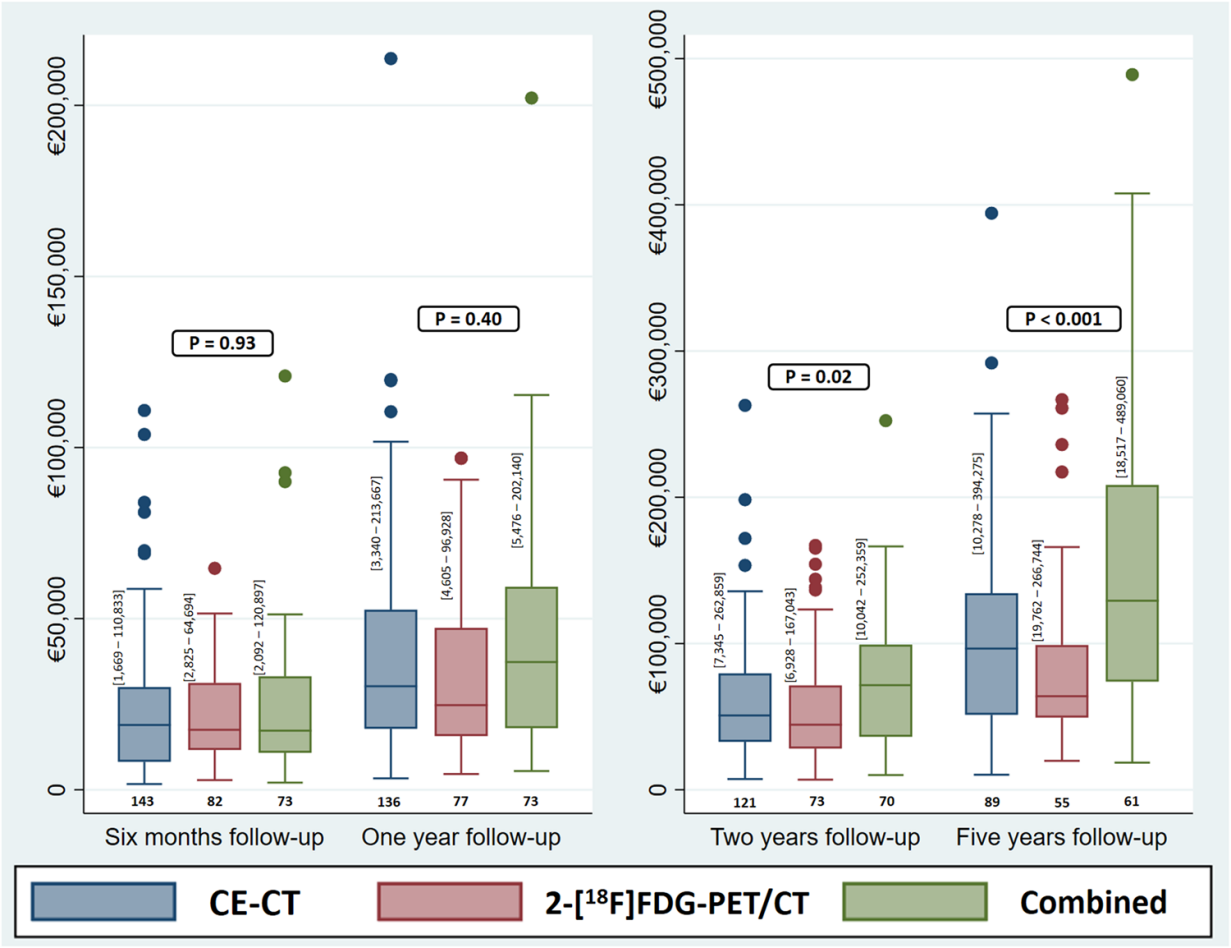
Box plots to compare accumulated costs for each study group during different follow-up periods. Patients needed to be alive for least three months, six months, one year, and two years to be included in the 6-month, 1-year, 2-year, and 5-year analyses, respectively. The numbers in brackets close to each box plot represent the range of cost in each specific group (CE-CT, contrast-enhanced computed tomography; FDG-PET/CT, ^18^Fluorodeoxyglucose positron emission tomography with integrated computed tomography).

A comparison of the accumulated costs for each study group showed no significant difference over the first two years of follow-up between the CE-CT group and the 2-[^18^F]FDG-PET/CT group, but the 2-[^18^F]FDG-PET/CT group had a considerably lower total cost over the first five years (Fig. 3). A comparison of the “cost per month” for each study group and for subgroups of patients with specific clinical characteristics is shown in Supplementary Material 2. The median cost per month for the 2-[^18^F]FDG-PET/CT group was €469 lower than that of the CE-CT group and €863 lower than that of the combined group (*P* = 0.39).

### Incremental cost-effectiveness ratio (ICER)

Table 4 shows the ICER for the 2-[^18^F]FDG-PET/CT group using CE-CT as the reference. The ICER for all patients was €-527/month, meaning that the total cost for 2-[^18^F]FDG-PET/CT group was €527 lower for each month of gained survival compared with the CE-CT group. The ICER remained negative after excluding patients from clinical trials and those diagnosed before 2009 (the starting point of 2-[^18^F]FDG-PET/CT use in our center). The ICER was also in favor of the 2-[^18^F]FDG-PET/CT group in patients with oligometastatic disease (− 617), de novo disease (− 690), or estrogen receptor-positive disease (-402). However, the ICER was in favor of the CE-CT group in patients with liver/lung metastases at baseline (ICER: 284) and patients with “ER-positive, HER2-negative, non-oligometastatic” disease (ICER: 153).

**Table 4:** Incremental cost-effectiveness ratio (ICER) for CE-CT and 2-[^18^F]FDG-PET/CT groups.

Characteristics	CE-CT			2-[^18^F]FDG-PET/CT			ICER***
	No. (%)	Survival*	Cost**	No. (%)	Survival*	Cost**	
All patients	144 (100)	30.0	91,547	83 (100)	44.3	83,965	− 527.9
Excluding patients from clinical trials	137 (95)	29.9	89,935	83 (100)	44.3	83,965	− 415.7
Excluding patients diagnosed before 2009	129 (90)	29.1	89,810	83 (100)	44.3	83,965	− 383.4
Patients with oligometastatic disease	18 (13)	40.3	103,996	8 (10)	94.0	70,847	− 617.3
ER-positive disease	118 (82)	34.8	88,835	69 (83)	46.5	84,122	− 402.8
HER2-negative disease	103 (72)	25.8	81,868	64 (77)	39.9	78,790	− 218.8
De novo metastatic breast cancer	31 (22)	32.8	98,716	17 (20)	56.6	82,291	− 690.1
Liver/lung metastases at baseline scan	92 (64)	26.6	84,917	46 (55)	45.9	90,402	284.0
Performance status at baseline < 2	111 (77)	29.1	93,971	66 (80)	45.9	89,879	− 243.3
Performance status at baseline ≥ 2	16 (11)	25.7	56,907	9 (11)	22	62,590	− 1547.5
ER + , HER2-negative, non-oligometastatic disease	98 (68)	30.0	83,969	58 (70)	44.3	86,161	153.2

CE-CT, contrast-enhanced computed tomography; 2-[^18^F]FDG-PET/CT, ^18^Fluorodeoxyglucose positron emission tomography with integrated computed tomography; ER, estrogen receptor; HER2, human epidermal growth factor receptor 2; ICER, incremental cost-effectiveness ratio.

*Median survival (month) for study group.

**Mean cost in Euro for study group.

***ICER shows the cost-efficacy of 2-[^18^F]FDG-PET/CT calculated as mean cost over median survival, using CE-CT as the reference.

A sensivitity analysis of median-based ICER for the 2-[^18^F]FDG-PET/CT group (median total cost: €65,685) resulted in − 556 using the CE-CT group as reference (median total cost: €73,667). The sensitivity analysis investigating the ICER for matched subgroups of patients from the CE-CT (n = 83) and 2-[^18^F]FDG-PET/CT groups is shown in Supplementary Material 3. The results were in line with the main analysis but showed a smaller difference (ICER: − 222).

## Discussion

Our results showed that the mean total cost per patient in the group of patients monitored with 2-[^18^F]FDG-PET/CT was €7582 lower than that for patients monitored with CE-CT. This difference was statistically insignificant, however, probably due to the large variation in the costs per patient. Using the CE-CT group as reference, the ICER for the 2-[^18^F]FDG-PET/CT group was €-527/month, indicating that response monitoring by 2-[^18^F]FDG-PET/CT results in gaining an extra month of survival at a lower cost (€527). In addition, 2-[^18^F]FDG-PET/CT was a more cost-effective modality than CE-CT for response monitoring MBC patients with favorable prognostic factors (oligometastatic or estrogen receptor-positive disease). In contrast, CE-CT was more cost-effective than 2-[^18^F]FDG-PET/CT in the subgroups of patients with unfavorable prognostic factors (performance status ≥ 2 or liver/lung metastases at diagnosis). Patients in the 2-[^18^F]FDG-PET/CT group had a higher number of short admissions (six vs. two) and fewer overnight hospital stays than patients in the CE-CT group (five vs. 12). The combined group (who were monitored with both modalities) had the highest total cost within the first year, two years, and five years, implying that comparions should be restricted to the CE-CT and 2-[^18^F]FDG-PET/CT groups.

As we know from our previous study, patients in the 2-[^18^F]FDG-PET/CT group had a lower median number of treatment lines (2 vs. 3, *P* = 0.005) and consequently fewer treatment changes during follow-up compared with the CE-CT group^7^. This may explain the lower total cost in the 2-[^18^F]FDG-PET/CT group as costs related to the treatment protocols represent the main share of the total cost. Therefore, the slightly increased cost of using 2-[^18^F]FDG-PET/CT could be balanced out over the longer follow-up period. Also, two previous studies show that 2-[^18^F]FDG-PET/CT can detect progression earlier than CE-CT and may lead to an earlier change of treatment^6,7^. This could explain the fewer overnight admissions in the 2-[^18^F]FDG-PET/CT group (and thus contribute to lower total costs) as re-admisions are often due to disease progression^25^. Another explanation could be the more recent diagnosis of patients in the 2-[^18^F]FDG-PET/CT group than the CE-CT group (2015 vs. 2013), allowing them to receive more advanced treatment protocols and thus potentially more short-stay admissions and fewer overnight hospital stays. On the other hand, the number of outpatient visits was almost same in the CE-CT and 2-[^18^F]FDG-PET/CT groups, in line with the similar follow-up period (29.7 vs. 30.1 months, respectively).

The ICER for the 2-[^18^F]FDG-PET/CT group was €284/month in the subgroup of patients with liver/lung metastases and €153/month for patients with “ER-positive, HER2-negative and non-oligometastatic” disease. The first subgroup is known to have a poor prognosis^26^, while the second subgroup is the most common clinical presentation for MBC patients^27^.

The first limitation of this observational study was its single-center and retrospective design, meaning that patients were not randomly allocated to the study groups. More patients in the CE-CT group received chemotherapy, while more patients in the 2-[^18^F]FDG-PET/CT group received CDK4/6 inhibitors at least once during the follow-up period. Also, more patients in the CE-CT group had liver/lung metastases at baseline scan than in the 2-[^18^F]FDG-PET/CT group (64% vs. 55%). Furthermore, the choice of imaging modality for response monitoring introduced an inherent limitation. The decision regarding which modality to use, whether CE-CT only, PET/CT only, or a combination of both, was made by the treating oncologist during the patient's initial visit. While all medical costs were fully covered by the national insurance system in Denmark, factors such as the oncologist's clinical judgment based on their experience, the patient's clinical condition, the specific organs involved, and patient preferences influenced this decision. While this retrospective study acknowledges the potential for bias in the modality selection process, a careful review of patients' baseline demographics and clinicopathological characteristics revealed no systemic bias in imaging modality selection. Notably, critical prognostic parameters were well-balanced between the main study groups.

Additional limitations pertain to the DRG pricing system, which is the only national pricing system used in Denmark healthcare^16,20^. The DRG/DAGS rates are based on an average cost of services that includes different patient groups, thus preventing a precise cost comparison between the study groups. The same approach was used for all study groups, however, which minimizes the risk of bias. Further, the Danish DRG system applies a single DRG rate for patients having several services during a single visit (e.g., if a patient has two outpatient visits in one day, only the most costly visit will have a DRG tariff). Imaging costs are included in the estimated costs related to the Department of Oncology, but specific differences between imaging modalities could not be estimated per patient as these costs are not listed separately during the admissions. We did not include costs related to additional visits in relation to a patient’s rehabilitation program, nor costs related to private palliative care centers outside of the hospital organization. Costs related to other resources outside of hospital were not considered in our analyses. Additionally, a longer follow-up period might be favorable as almost 30% of included patients were still alive at the end of the study, which could bias the long-term analysis. Lastly, we acknowledge that the retrospective design of our study limited our ability to perform a QALY-based cost-effectiveness analysis, as it necessitates prospective data collection for quality of life assessments. Therefore, this important aspect was not included in our evaluation.

We believe this to be the first cost-effectiveness analysis of 2-[^18^F]FDG-PET/CT for response monitoring of MBC patients. A strength of the study was the relatively large number of patients (88% of eligible patients) representative of daily clinical practice, which allowed us to do a cost-effectiveness analysis based on the routine management of MBC patients. The patients had equal access to healthcare services that were covered by the same national insurance system^15^. Lastly, we compared inpatient versus outpatient costs and short-term versus long-term costs to better understand the cost-efficacy elements of the imaging modalities.

Some previous studies have investigated the cost-efficacy of using PET/CT at early stages of breast cancer or for detection of recurrence. A pilot cost-effectiveness study compared the cost/quality-adjusted life year (QALY) in three countries (US, UK, and Netherlands) among stage II/III breast cancer patients and showed that using 2-[^18^F]FDG-PET/CT as a screening modality to detect distant metastases may result in incremental QALY gains in all countries. They concluded that 2-[^18^F]FDG-PET/CT was a cost-effective modality in the Netherlands and US, but not in the UK due to varying costs of services and different healthcare policies^28^. The average lifetime hospital-based costs of patients with advanced breast cancer was reported to be around €53,000 in the Netherlands^29^. The Dutch Council for Public Health and Health Care has set an informal ceiling ratio of €80,000 per gained extra year of survival for cancer patients, which could grant the use of 2-[^18^F]FDG-PET/CT for response monitoring of MBC patients^10,30^.

An economic evaluation of using PET/CT to detect recurrence of breast cancer was conducted with limited data (available up to 2010) and found PET/CT unlikely to be cost-effective in the detection of recurrence^31^. These results could be expected as 2-[^18^F]FDG-PET/CT cannot be cost-effective in the short-term—we observed favorable cost-effectiveness for CE-CT for the first three months of follow-up.

A study on the application of PET/CT with FES and ^89^Zr-trastuzumab in hypothetical cohorts of MBC patients for the decision of first-line hormonal and trastuzumab therapies suggested a potential cost-effectiveness of these modalities compared with using pathology of biopsies as ususal care. The authors concluded that improved sensitivity and specificity by FES-PET/CT and ^89^Zr-trastuzumab-PET/CT may result in prolonged progression-free and overall survival and thus increase potential cost-effectiveness^10^. This is in line with our results as we observed better overall survival in the 2-[^18^F]FDG-PET/CT group compared with the CE-CT group^7^, possibly explaining the better cost-effectiveness for 2-[^18^F]FDG-PET/CT in the long-term (Fig. 3). Moreover, it is noteworthy that even when we examined the "pure" groups of CE-CT and 2-[^18^F]FDG-PET/CT, excluding patients who had received the opposite modality once, our sensitivity analysis revealed even greater differences in survival outcomes between the CE-CT and 2-[^18^F]FDG-PET/CT groups^7^, indicating the potential cost-effectiveness of the 2-[^18^F]FDG-PET/CT modality as the primary response monitoring tool for MBC patients.

The overall economic burden of MBC is expected to increase due to the rising number of women living with the disease^32,33^. A more accurate response monitoring modality could reduce healthcare costs through improvements in clinical management^34^. Our results suggest 2-[^18^F]FDG-PET/CT as a cost-effective modality based on ICER assessments, but other clinical and logistical considerations need to be taken into account^35^. Overall, PET/CT has proved to be a promising strategy to reduce the cost of cancer management by potentially avoiding side effects or earlier termination of expensive, ineffective anti-cancer treatments^7,36^. Any change in patient management that helps to avoid ineffective medical treatment is essential for improved health outcomes^37^.

The average lifetime cost of managing MBC patients is estimated to be around €94,000 in Sweden^38^, and this is expected to increase due to treatment landscape improvement and higher cancer-related drug costs^39^. Compared with 2019, the current price difference in Denmark between a 2-[^18^F]FDG-PET/CT scan (€1262) and a CE-CT scan (€324) is much lower^40^, making it even more reasonable to investigate the efficacy of 2-[^18^F]FDG-PET/CT in prospective clinical settings. The indication of 2-[^18^F]FDG-PET/CT for response monitoring in patients with MBC requires a better understanding of its cost-effectiveness, which could be provided by prospective randomized trials of 2-[^18^F]FDG-PET/CT as an intervention modality in a clinical, multi-center approach. Future studies should aim to incorporate quality of life assessments and QALY-based calculations to offer a more comprehensive evaluation of the cost-effectiveness and overall impact of 2-[^18^F]FDG-PET/CT in the context of metastatic breast cancer management.

## Conclusion

This single-center observational study indicated that 2-[^18^F]FDG-PET/CT is a more cost-effective modality than CE-CT for response monitoring in patients with metastatic breast cancer. We found that 2-[^18^F]FDG-PET/CT was more cost-effectiveness in subgroups of patients with favorable prognostic factors (oligometastatic or estrogen receptor-positive disease), while CE-CT was more cost-effective in patients with unfavorable prognostic factors (liver/lung metastases). The use of 2-[^18^F]FDG-PET/CT for response monitoring in patients with metastatic breast cancer could improve patient survival and save the healthcare system valuable resources, but this needs to be confirmed in prospective randomized trials.

### Supplementary Information

Supplementary Table 1.

Supplementary Table 2.

Supplementary Table 3.

## Data Availability

The datasets generated during the current study are available from the corresponding author on reasonable request.
