# Supplementary material for: Cost-effectiveness of 2-[18F]FDG-PET/CT versus CE-CT for response monitoring in patients with metastatic breast cancer: a register-based comparative study
Source: Sci Rep. 2023 Sep 28;13:16315. doi: 10.1038/s41598-023-43446-7 (PMC10539314; doi:10.1038/s41598-023-43446-7)
Supplement: Supplementary file 1 — Supplementary Table 1. [file 41598_2023_43446_MOESM1_ESM.docx]

| **Supplementary Material 1.** Summary of prognostic parameters and survival analysis by study group | | | | | |
| --- | --- | --- | --- | --- | --- |
| **Characteristics** | | **Study Groups** | | | **P-value** |
|  |  | **CE-CT**  (*n* = 144) | **2-[^18^F]FDG-PET/CT**  (*n* = 83) | **Combined**  (*n* = 73) |  |
| **Important prognostic parameters^*^** | Year of inclusion | 2013 (2007-2017) | 2015 (2009-2018) | 2013 (2007-2017) | <0.001 |
|  | Age at inclusion (years) | 66.1 (38.2-84.5) | 64.7 (28.2-86.7) | 60.4 (31.0-95.3) | 0.004 |
|  | Performance status ≥2 at diagnosis | 16 (11.1) | 9 (10.8) | 1 (1.4) | 0.02 |
|  | ER-positive | 118 (81.9) | 69 (83.1) | 55 (75.3) | 0.24 |
|  | HER2-positive disease | 13 (9.0) | 8 (9.6) | 18 (24.7) | 0.02 |
|  | ER-negative & HER2-negative | 12 (8.3) | 8 (9.6) | 5 (6.9) | 0.80 |
|  | De novo metastatic breast cancer | 31 (21.5) | 17 (20.5) | 20 (27.4) | 0.56 |
|  | Oligometastatic disease^**^ | 18 (12.5) | 8 (9.6) | 15 (20.6) | 0.14 |
|  | Liver/lung metastases at baseline | 92 (63.9) | 46 (55.4) | 36 (49.3) | 0.10 |
|  | Number of involved organ at baseline scan | | | | |
|  | Single organ | 42 (29.2) | 13 (15.7) | 22 (30.1) | 0.06 |
|  | Two organs | 101 (70.1) | 68 (81.9) | 49 (67.1) |  |
|  | Five organs | 1 (0.7) | 2 (2.4) | 2 (2.7) |  |
| **Survival analyses^***^** | Mortality during follow-up | 123 (85.4) | 45 (54.2) | 47 (64.4) | <0.001 |
|  | Survival time (months) | 30.0 (25.5-36.0) | 44.3 (29.7-80.2) | 54.0 (44.3-75.6) | <0.001 |
|  | Survival probabilities (%) | | | | |
|  | 1-year survival | 84.7 (77.7-89.7) | 91.6 (83.1-95.9) | 95.9 (87.8-98.7) | 0.03 |
|  | 2-year survival | 63.9 (55.5-71.1) | 69.3 (58.0-78.1) | 87.7 (77.6-93.4) | 0.001 |
|  | 5-year survival | 15.7 (9.7-23.0) | 65.5 (28.9-54.1) | 43.2 (30.4-55.3) | <0.001 |
|  | 10-year survival | - | 8.5 (7.4-39.0) | 23.7 (13.1-36.1) | <0.001 |
| CE-CT: contrast-enhanced computed tomography, 2-[^18^F]FDG-PET/CT: ^18^Fluorodeoxyglucose positron emission tomography with integrated computed tomography; ER: estrogen receptor; HER2: human epidermal growth factor receptor 2  ^*^Data shown as median (interquartile range) and frequency (%).  ^**^Oligometastatic cancer refers to patients with fewer than five metastatic lesions in a single organ.  ^***^Data shown as median (95% confidence interval). | | | | |  |
