# Supplementary material for: Cost-effectiveness of 2-[18F]FDG-PET/CT versus CE-CT for response monitoring in patients with metastatic breast cancer: a register-based comparative study
Source: Sci Rep. 2023 Sep 28;13:16315. doi: 10.1038/s41598-023-43446-7 (PMC10539314; doi:10.1038/s41598-023-43446-7)
Supplement: Supplementary file 2 — Supplementary Table 2. [file 41598_2023_43446_MOESM2_ESM.docx]

| **Supplementary Material 2.** Comparison of overall median total costs per month by study group^*^ | | | | | | |
| --- | --- | --- | --- | --- | --- | --- |
| **Characteristics** | **Study groups (*N* = 300*)*** | | | | | |
|  | **CE-CT** (*n* = 144*)* | | **2-[^18^F]FDG-PET/CT** (*n* = 83*)* | | **Combined** (*n* = 73*)* | |
|  | No. | Total cost per month | No. | Total cost per month | No. | Total cost per month |
| All patients | 144 | 3,055 (558-12,433) | 83 | 2,586 (285-8,943) | 73 | 3,449 (309-7,525) |
| Excluding patients from clinical trials | 129 | 3,053 (558-12,433) | 83 | 2,586 (285-8,943) | 60 | 3,380 (309-7,525) |
| Excluding patients diagnosed before 2009 | 137 | 3,062 (558-12,433) | 83 | 2,586 (285-8,943) | 65 | 3,484 (309-7,525) |
| Patients with oligometastatic disease | 18 | 2,506 (918-5,325) | 8 | 1,507 (285-3,820) | 15 | 3,272 (309-5,521) |
| De novo metastatic breast cancer | 31 | 3,107 (696-9,219) | 17 | 3,111 (285-8,176) | 20 | 3,617 (593-5,403) |
| Estrogen receptor-positive disease | 118 | 2,659 (558-12,433) | 69 | 2,252 (599-7,271) | 55 | 2,822 (309-6,977) |
| HER2-negative disease | 103 | 2,746 (558-12433) | 64 | 2,393 (599-8,494) | 44 | 3,225 (309-7,525) |
| Performance status at baseline < 2 | 111 | 3,058 (595-12,433) | 66 | 2,541 (285-8,494) | 53 | 2,831 (309-7,525) |
| Performance status at baseline ≥ 2 | 16 | 2,849 (558-7,951) | 9 | 3,542 (647-7,198) | 1 | ^-^ |
| CE-CT: contrast-enhanced computed tomography, 2-[^18^F]FDG-PET/CT: ^18^fluorodeoxyglucose positron emission tomography with integrated computed tomography  ^*^Data are shown as median (range) and in Euros. Median cost per month was calculated by dividing the total cost by follow-up time. | | | | | | |
