# Supplementary material for: Cost-effectiveness of 2-[18F]FDG-PET/CT versus CE-CT for response monitoring in patients with metastatic breast cancer: a register-based comparative study
Source: Sci Rep. 2023 Sep 28;13:16315. doi: 10.1038/s41598-023-43446-7 (PMC10539314; doi:10.1038/s41598-023-43446-7)
Supplement: Supplementary file 3 — Supplementary Table 3. [file 41598_2023_43446_MOESM3_ESM.docx]

| **Supplementary Material 3.** Cost per month (adjusted to 2019 level) and incremental cost-effectiveness ratio (ICER) for the subgroup of CE-CT patients (n = 83) from CE-CT group (matched to 2-[^18^F]FDG-PET/CT group) and the 2-[^18^F]FDG-PET/CT group. | | | | | | | | | |
| --- | --- | --- | --- | --- | --- | --- | --- | --- | --- |
| **Characteristics** | **CE-CT** | | | | **2-[^18^F]FDG-PET/CT** | | | | **ICER^****^** |
|  | No. | Survival**^*^** | Cost (€) | | No. | Survival**^*^** | Cost (€) | |  |
|  |  |  | Per month**^**^** | Total**^***^** |  |  | Per month**^**^** | Total**^***^** |  |
| All patients | 83 | 30.0 | 3,242 | 87,155 | 83 | 44.3 | 2,586 | 83,965 | -222.1 |
| Excluding patients from clinical trials | 73 | 30.0 | 3,253 | 88,803 | 83 | 44.3 | 2,586 | 83,965 | -336.8 |
| Excluding patients diagnosed before 2009 | 82 | 29.1 | 3,177 | 87,022 | 83 | 44.3 | 2,586 | 83,965 | -200.5 |
| Patients with oligometastatic disease | 9 | 33.9 | 2,853 | 107,451 | 8 | 94.0 | 1,507 | 70,847 | -609.1 |
| Estrogen receptor-positive disease | 67 | 33.1 | 2,746 | 86,703 | 69 | 46.5 | 2,252 | 84,122 | -193.0 |
| HER2-negative disease | 65 | 29.1 | 3,088 | 79,238 | 64 | 39.9 | 2,393 | 78,790 | -41.6 |
| De novo metastatic breast cancer | 21 | 30.1 | 3,199 | 97,724 | 17 | 56.6 | 3,111 | 82,291 | -583.3 |
| Liver/lung metastases at baseline scan | 52 | 25.2 | 2,844 | 76,956 | 46 | 45.9 | 3,677 | 90,402 | 648.9 |
| Performance status at baseline < 2 | 61 | 29.1 | 3,089 | 91,604 | 66 | 45.9 | 2,541 | 89,879 | -102.6 |
| Performance status at baseline ≥ 2 | 11 | 27.9 | 2,610 | 55,670 | 9 | 22 | 3,542 | 62,590 | -1166.0 |
| CE-CT: contrast-enhanced computed tomography; 2-[^18^F]FDG-PET/CT: ^18^fluorodeoxyglucose positron emission tomography with integrated computed tomography; ICER: incremental cost-effectiveness ratio  ^*^Median overall cost calculated through total costs adjusted to the follow-up time.  ^**^Median survival (month) for study group.  ^***^Mean overall cost for study group.  ^****^ICER shows the cost-efficacy of 2-[^18^F]FDG-PET/CT calculated as mean cost over median survival, using CE-CT as the reference | | | | | | | | | |
